# The development of whole blood titanium levels after instrumented spinal fusion – Is there a correlation between the number of fused segments and titanium levels?

**DOI:** 10.1186/1471-2474-13-159

**Published:** 2012-08-27

**Authors:** Ingmar Ipach, Ralf Schäfer, Falk Mittag, Carmen Leichtle, Petra Wolf, Torsten Kluba

**Affiliations:** 1 Department of Orthopaedic Surgery, University Hospital Tuebingen, Tuebingen, Germany; 2 Technical University of Munich, Department of Medical Statistics and Epidemiology, Ismaninger Str. 22, Munich, 81675, Germany

**Keywords:** Spinal fusion, Titanium serum levels, Interbody devices, Pedicle screws

## Abstract

**Background:**

Most modern spinal implants contain titanium and remain in the patient’s body permanently. Local and systemic effects such as tissue necrosis, osteolysis and malignant cell transformation caused by implants have been described. Increasing tissue concentration and whole blood levels of ions are necessary before a disease caused by a contaminant develops. The aim of the present study was the measurement of whole blood titanium levels and the evaluation of a possible correlation between these changes and the number of fused segments.

**Methods:**

A prospective study was designed to determine changes in whole blood titanium levels after spinal fusion and to analyze the correlation to the number of pedicle screws, cross connectors and interbody devices implanted.

Blood samples were taken preoperatively in group I (n = 15), on the first, second and 10^th^ day postoperatively, as well as 3 and 12 months after surgery.

Group II (n = 16) served as a control group of volunteers who did not have any metal implants in the body. Blood samples were taken once in this group.

The number of screw-rod-connections and the length of the spinal fusion were determined using radiographic pictures. This study was checked and approved by the ethical committee of the University of Tuebingen.

**Results:**

The mean age in group I was 47 ± 22 years (range 16 - 85 years). There were three male (20%) and twelve female (80%) patients. The median number of fused segments was 5 (range 1 to 11 segments).

No statistically significant increase in the titanium level was seen 12 months after surgery (mean difference: -7.2 μg/l, 95% CI: -26.9 to 12.5 μg/l, p = 0.446). By observing the individual titanium levels, 4 out of 15 patients demonstrated an increase in titanium levels 12 months after surgery.

No statistically significant correlation between fused segments (r = -0.188, p = 0.503) length of instrumentation (r = -0.329, p = 0.231), number of interbody devices (r = -0.202, p = 0.291) and increase of titanium levels over the observation period was seen.

**Conclusions:**

Instrumented spinal fusion does not lead to a statistically significant increase in whole blood titanium levels. There seems to be no correlation between the number of pedicle screws, cross connectors and interbody devices implanted and the increase of serum titanium levels.

## Background

Spinal surgery -especially instrumented spinal fusion- has increasing importance in treatment of patients with chronic low back pain caused by osteoligamentary instability, degenerative disc diseases or spondylolisthesis. Most of the modern spinal implants contain titanium and remain in the patient’s body permanently. Little is known about the influence of free metal ions on a patient’s health. Negative effects such as tissue necrosis, osteolysis and malignant cell transformation have been described [1].

Rising tissue concentration and serum levels of ions are necessary before a disease caused by a contaminant develops [2,3]. It has been shown that metal ions such as vanadium and cobalt chromium decrease monocyte and macrophage survival in a dose-dependent manner [4].

It has also been shown that titanium-alloy particles stimulate cytokine release. Additionally opsonization of machined particles with human serum increased the release of macrophages and cytokines [5].

Activation of the monocyte-macrophage-system leads to a local inflammatory reaction and is a relevant promoter for prosthetic loosening [6].

Although titanium is characterized by excellent qualities, there are many indications that it is not inert. Nephrotoxicity, cardiac and hepatic injury have been described after oral administration to rats [7]. Patients undergoing revision for non-fusion after posterior lumbar spondylodesis, were observed to have a high paraspinal tissue concentration of titanium [8], and rising serum titanium levels after spinal fusion with a correlation to the amount of fused segments has been demonstrated [9]. Free titanium particles lead to an increase in prostaglandin and interleukin synthesis as an indicator of an inflammatory process with a negative influence on bone formation [10,11].

Corrosion of titanium ions in the surrounding tissue and the movement of these ions into the distant organs may cause systemic problems. By using rat peritoneal macrophages and by determining metal concentration in sheep after spinal fusion, an accumulation in the different organs especially in the brain, lungs, liver, spleen, kidneys and lymph nodes was detected. Inflammation and osteolysis was seen in the macrophages [12-16]. On the other hand, titanium is present as a micronutrient in the body even without any metal implants. A normal value for titanium in blood seems to be between 50-150 ug/l (Synlab/Leinfelden, Germany).

The aim of the present study was to detect a possible increase in titanium levels after spinal fusion. We also wanted to investigate a possible correlation between the number of implanted pedicle screws, cross connectors and interbody devices on titanium levels.

## Methods

A prospective study was performed to detect the effects of spinal fusion on titanium levels. For instrumentation, the Muenster-Anterior/Posterior-Doublerod-System® (Schäfer. Micomed, Schorndorf, Germany) and the Expedium® (Depuy-spine Raynham, Massachusetts, USA) systems were used.

Group I patients (n = 15) underwent instrumented spinal fusion in a period between January 2006 and March 2009. Blood samples were taken preoperatively, on the first, second and 10^th^ day postoperatively, as well as 3 and 12 months after surgery (Table 1).

**Table 1:** Demographic data group I

**Patient**	**sex**	**age**	**Diagnosis**	**Fused levels**	**length of intstrumantation**	**Number of interbody devices**	**Type of Implant**	**Titanium levels (ug/l)**				
								**Präoperativ**	**2.p.o.**	**10.p.o.**	**3 Mon p.o.**	**12 Mon p.o.**
1	f	73	DLS	10	1626	3	MPDS	56	34	26	29	37
2	f	55	LS	1	381	1	MPDS	33	44	ø	63	36
3	f	16	Scoliosis	6	864	0	MADS	34	ø	48	ø	31
4	f	16	Scoliosis	6	869	0	MADS	39	43	39	ø	96
5	f	22	Scoliosis	5	811	0	MADS	48	ø	42	54	94
6	m	45	Scoliosis	5	1076	2	MADS	54	42	44	55	31
7	f	68	LS	1	370	1	Expedium	53	ø	39	54	33
8	f	85	LSCS	4	824	1	Expedium	61	49	49	63	96
9	f	61	LS	1	410	1	Expedium	53	ø	49	40	47
10	f	61	LSCS	2	550	1	Expedium	49	ø	31	45	35
11	m	30	LS	1	405	1	Expedium	47	52	ø	40	142
12	f	42	Scoliosis	6	965	0	MADS	38	ø	ø	37	42
13	f	20	Scoliosis	11	1520	0	Expedium	51	42	3	38	37
14	f	55	DLS	6	1415	0	Expedium	69	33	37	32	36
15	m	58	DLS	4	540	0	Expedium	34	34	45	82	34

*DLS*: Degenerativ lumbar scoliosis, *LS*: lumbar spondylolisthesis, *LSCS*: lumbar spinal canal stenosis, *MAPS*: Muenster-Anterior-doublerod-system, *MPDS*: Muenster-posterior-doublerod-system.

To evaluate a possible influence of lifetime exposure on titanium levels, a control group (group II) was created. Group II (n = 16) consisted of volunteers who did not have any metal implants in the body and were statistically significantly younger than group I. Blood samples were taken once in this group (Table 2).

**Table 2:** Demographic data group II

**Patient**	**sex**	**age**	**Titanium levels (ug/l)**
1	f	26	59
2	m	55	47
3	m	33	30
4	f	24	37
5	f	55	33
6	m	24	35
7	m	31	57
8	f	50	59
9	f	28	58
10	f	29	55
11	f	50	56
12	m	29	61
13	f	24	33
14	f	24	53
15	f	20	58
16	m	30	59

Only patients with a normal liver and kidney function and no previous history of metal implants were included in the present study.

**Figure 1:**
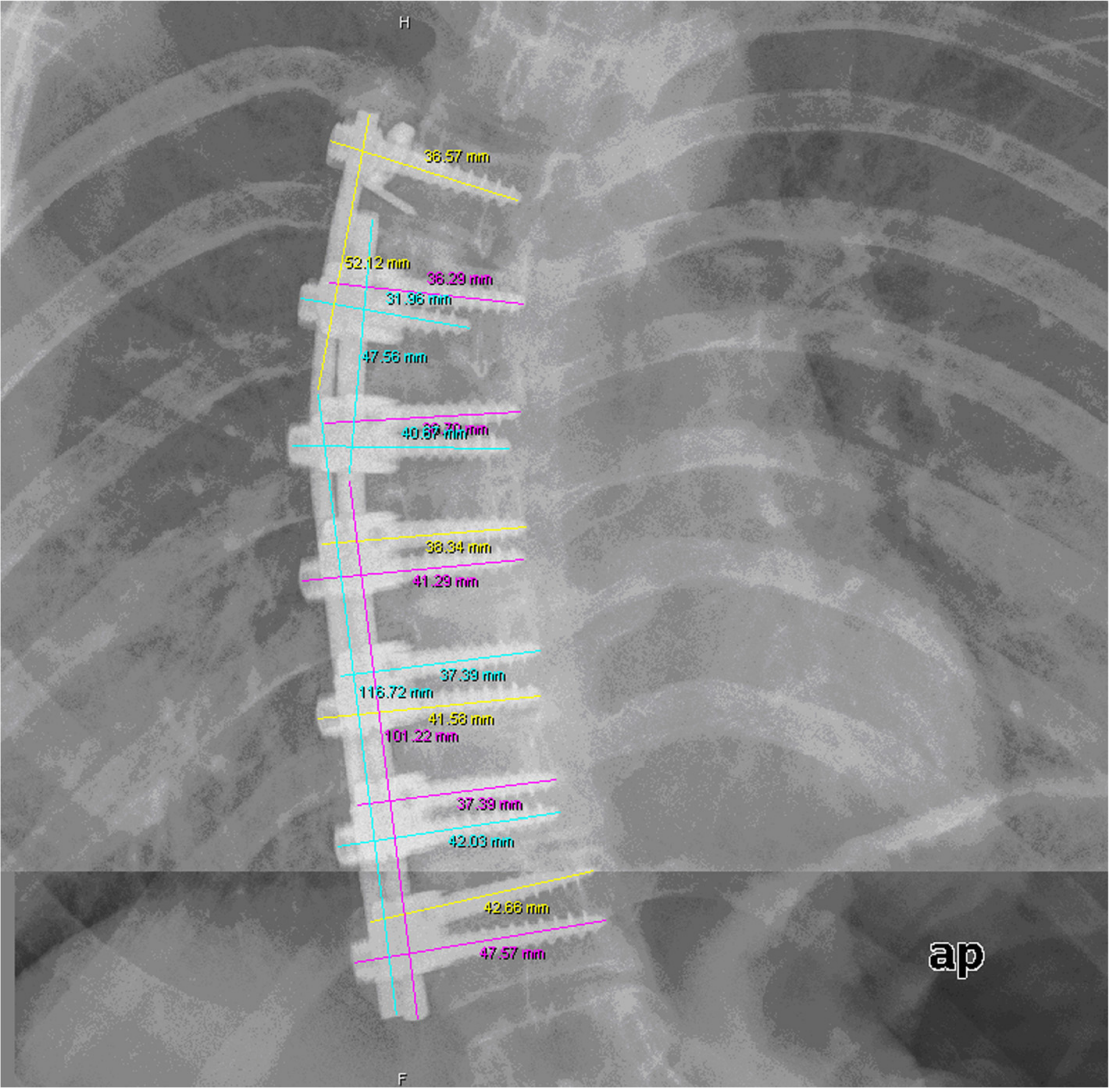
**Technique of length-measurement of the spinal fusion on lateral radiographs of the spine.** The length of each screw and rod were added to obtain the total length of spinal fusion.

**Figure 2:**
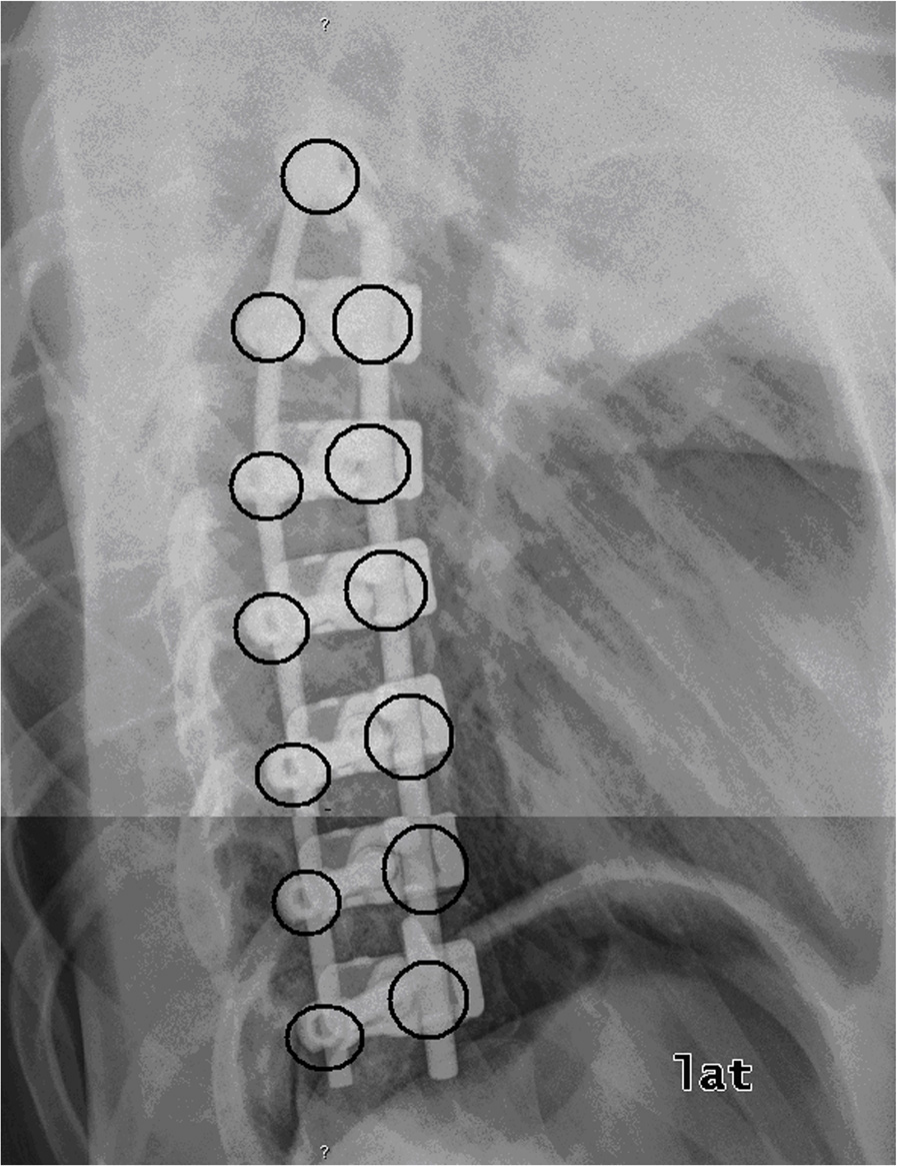
Technique for counting the different connections on a.p. radiographs of the spine.

The number of screw-rod-connections and the length of the spinal fusion were determined using radiographic pictures (Figure 1, 2).

### Study registration and informed consent

This study was checked and approved by the local ethical committee of the University of Tuebingen. Written informed consent was obtained from all patients to take part in this study.

#### Blood samples and titanium analysis

Blood samples were taken by using titanium-free canulas and EDTA-tubes.

Collected blood was stored at 5°C until measurement. Titanium was measured by ICP-MS (inductively coupled plasma mass spectrometry) (Synlab/Leinfelden, Germany). Therefore titanium was ionized through plasma at a temperature of 5000°C. Depending on their mass, ions were separated and counted using a spectrometer. Detection limit of titanium ions was >0.02 parts per trillion.

Normal values for whole blood titanium levels were in a range between 50-150 ug/l in blood-samples.

#### Statistical analysis

Categorical variables are presented as frequencies and percentages, and continuous variables as means and standard deviations, or medians and ranges. A paired *t*-test was used to evaluate the difference in titanium levels preoperatively and 12 months after surgery. To compare the two groups, independent sample t tests or exact Mann Whitney *U* test were used. Differences are presented with 95% confidence intervals (CI). A relationship of titanium levels with fused segments, length of instrumentation and number of interbody devices was evaluated using the Spearman correlation coefficient.

All reported P values are two-sided, with a significance level of 0.05 and have not been adjusted for multiple testing. All analyses were performed with the use of R 2.13.2 (R Foundation for Statistical Computing, Vienna, Austria) and IBM SPSS 20.

## Results

The mean age in group I was 47 ± 22 years (range 16 - 85 years). There were three male (20%) and twelve female (80%) patients. The median number of fused segments was 5 (range 1 to 11 segments). Demographic data of this group are shown in Table 1.

The whole blood titanium levels in group I before and in the postoperative period are shown in Table 3.

**Table 3:** Whole blood titanium levels in group I before and in the postoperative period

**time**	**mean titanium level (μg/l)**	**n**	**standard deviation**	**minimum titanium level (μg/l)**	**maximum titanium level (μg/l)**
before surgery	47.9	15	10.6	33.0	69.0
2 th day after surgery	41.4	9	6.7	33.0	52.0
10^th^ day after surgery	41.5	12	7.5	26.0	49.0
3 months after surgery	48.6	13	15.0	29.0	82.0
12 months after surgery	55.1	15	34.4	31.0	142.0

**Figure 3:**
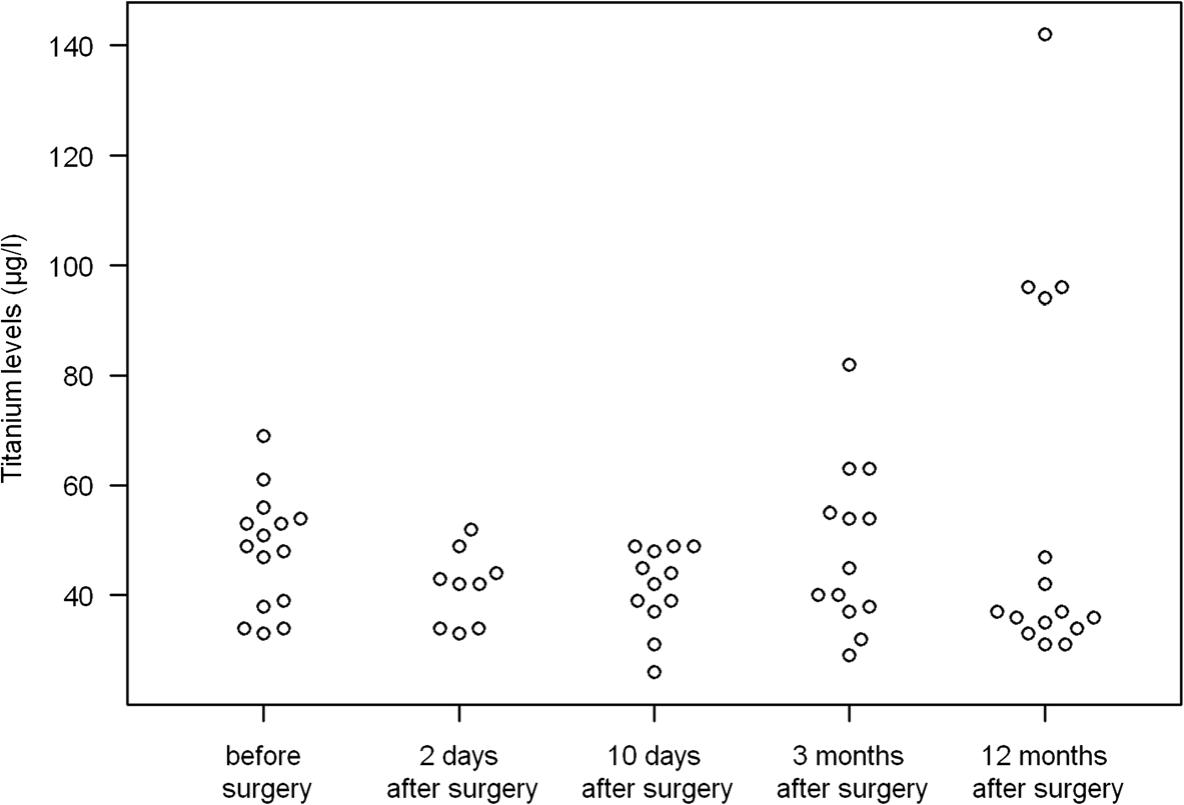
Development of titanium levels after spinal fusion in group I.

**Figure 4:**
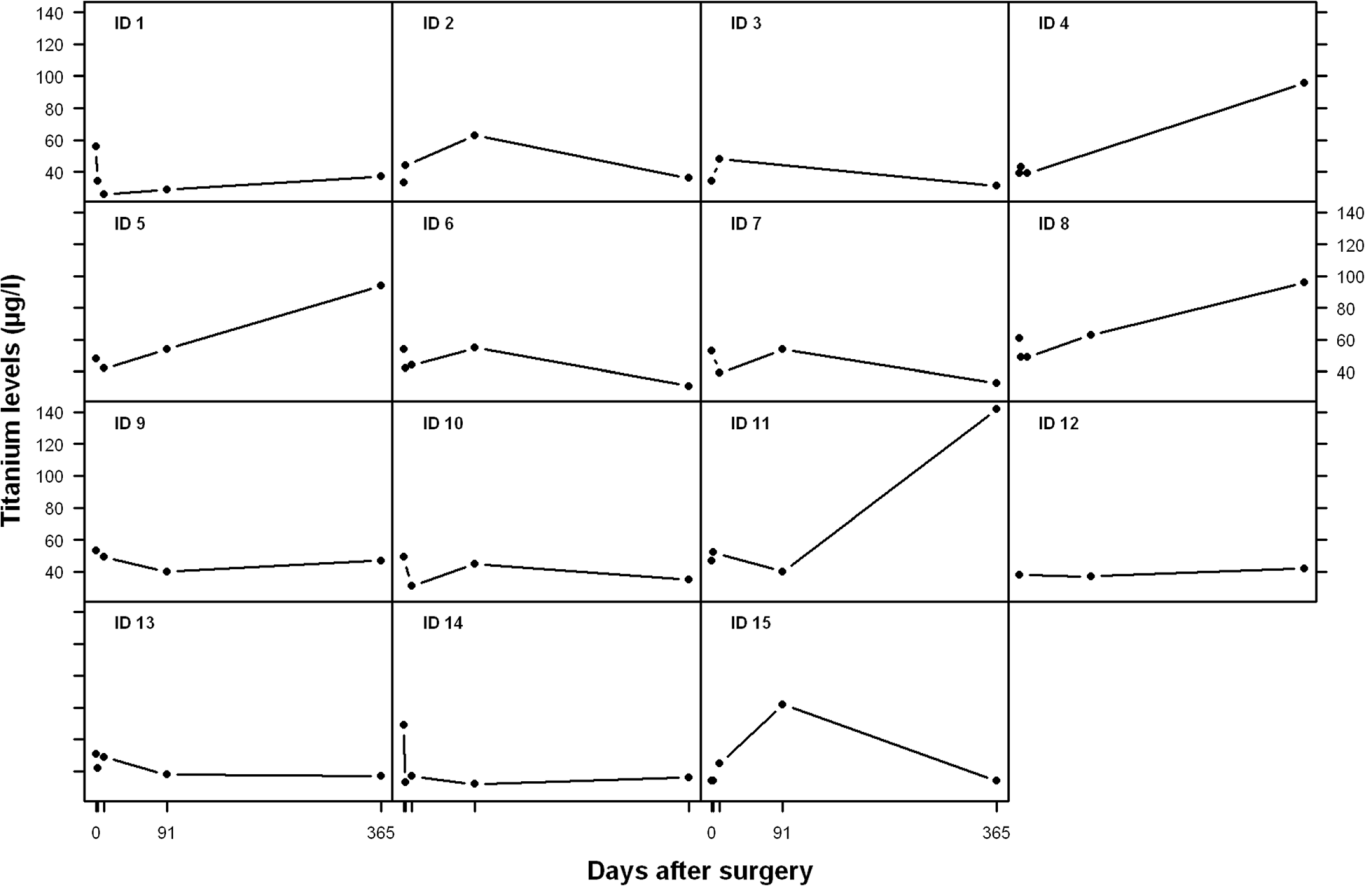
Individual development of titanium levels in the different patients.

No statistically significant increase in titanium levels was seen 12 months after surgery (mean difference: -7.2 μg/l, 95% CI: -26.9 to 12.5 μg/l, p = 0.446) (Figure 3). 4 out of 15 patients demonstrated an increase in titanium levels 12 months after surgery (ID 4,5,8,11)(Figure 4). 8 out of 15 patients presented with sub-normal levels of titanium.

Titanium levels were evaluated in all patients before surgery and 12 months after surgery. Titanium levels were evaluated in 9 patients (out of 15) two days after surgery, 10 days after surgery in 12 patients (out of 15) and 3 months after surgery in 13 patients (out of 15). 11 patients presented sub-normal titanium levels12 months after surgery.

One patient had to be revised due to a loss of correction 6 months after primary surgery, and re-instrumentation was necessary. The development of titanium levels of this patient is also shown in Table 1 (ID 1).

The mean age in group II was 33 ± 12 years (range 20 – 55 years). There were 6 male and 10 female patients in this group. The mean titanium level in this group was 49.4 ± 11.5 μg/l (range 30 –61 μg/l). There was a statistically significant difference regarding age between the two groups (mean difference: 14 years, 95% CI: 0.5 to 27.2 years, p = 0.042).

When comparing preoperative titanium levels in group I with group II, there was no statistically significant difference (p = 0.464).

No statistically significant correlation between the number of fused segments (r = -0.188, p = 0.503), length of instrumentation (r = -0.329, p = 0.231), number of interbody devices (r = -0.202, p = 0.291) and development of titanium levels over the observation period was seen.

## Discussion

Titanium as a light and strong material is often used as an alloy in orthopedic implants, and is generally regarded as safe for the organism.

Titanium is present as a micronutrient in the body even without metal implants, and most is stored intracellularly. We therefore analyzed not only serum titanium levels as done in previous studies [9,17], but the total amount of titanium in the blood.

Titanium alloy used in pedicle screws and interbody devices contained approximately 90% titanium, 6% aluminum and 4% vanadium [18-20]. Therefore an evaluation of titanium levels in patients after instrumented spinal fusion might be interesting with regard to possible effects caused by metal particles and for early detection of none-fusions.

In the present study, no statistically significant raise in titanium levels during the observation period after spinal fusion was seen. In contrast to our findings, previous studies demonstrated an increase in titanium levels [9,17]. One possible explanation for the different results might be the use of different samples for the evaluation of titanium levels. As mentioned above, we used whole blood samples in the present study as most titanium is present in macrophages. Travis et al. [9] and Kasai et al. [17] analyzed free titanium ions in serum.

Interestingly, when observing the individual development of whole blood titanium levels, 4 out of 15 patients demonstrated an increase in titanium levels 12 months after surgery. It is unclear why there was such a spread in titanium levels 12 months after surgery. The distribution of metal ions after spinal fusion seems to be a multifactorial process which has not been evaluated. As we analyzed whole blood samples, the total amount of titanium was evaluated (free and intracellular titanium ions). These findings support previous findings by Richardson et at [9] who also stated that the individual’s biological response and elimination of titanium ions might be extremely variable. Another possible explanation might be that these four individuals have undergone unknown environmental exposure. Another possible reason for the increase in titanium levels in these individuals might be a reduced renal clearance, but all patients demonstrated normal renal function during the observation period. Individual titanium elimination might also explain why 8 out of 15 patients presented with sub-normal levels of titanium before surgery, and 10 out of 15 patients 12 months after surgery.

Initial titanium levels seem to have no effect on postoperative titanium levels. The fact that the probands in group II were younger than the patients in group I does not support the theory of continuously rising of levels during a lifetime caused by a longer environmental exposure time.

There was no correlation between the number of pedicle screws, cross connectors and interbody devices which support the findings by Travis et al. [9] who also found no correlation. One possible explanation for this finding might be that there is a balance between absorption from the alloy and renal elimination.

One of the main strengths of the present study is the use of whole blood samples. To our knowledge, this is the first study evaluating titanium levels after spinal fusion in whole blood. As demonstrated by Mohammed Bakir [16], there is strong contact between monocytes and the titanium surface with a possible absorption of titanium particles. This might explain the different standard values for titanium in serum (<7 ug/l) and whole blood 50-150 ug/l). By using whole blood samples, total amount of titanium and not only free ions could be detected.

We also note several limitations of the present study. First, the whole titanium levels at the second and 10^th^ postoperative day are incomplete, caused by a sample error. The results for this point of time might therefore not be meaningful.

We did not differentiate between the two spinal implant systems used when analyzing serum titanium levels. This might also be of interest and should be evaluated in further studies. Because of the small sample numbers, there may be low power with a possible type II error.

## Conclusion

Finally, there seems to be no increase in serum titanium levels after spinal fusion. Whether the trend is stable, or if whole titanium levels increase in patients with non-fusion or after a period of 12 months needs to be investigated in further observations.

## Competing interests

The authors declare that they have no competing interests.

## Authors’ contributions

II: participated in the design of the study, drafted the manuscript, RS: collecting data, FM: participated in the of the study, collecting data, CL: performed spinal surgery, PW: performed statistical analysis, TK: conceived of the study, and participated in its design and coordination. All authors read and approved the final manuscript.

## Pre-publication history

The pre-publication history for this paper can be accessed here:

http://www.biomedcentral.com/1471-2474/13/159/prepub
